# Re-emerging concepts of immune dysregulation in autism spectrum disorders

**DOI:** 10.3389/fpsyt.2022.1006612

**Published:** 2022-10-19

**Authors:** Alina Erbescu, Sorina Mihaela Papuc, Magdalena Budisteanu, Aurora Arghir, Monica Neagu

**Affiliations:** ^1^Victor Babes National Institute of Pathology, Bucharest, Romania; ^2^Faculty of Biology, Doctoral School, University of Bucharest, Bucharest, Romania; ^3^Prof. Dr. Alex. Obregia Clinical Hospital of Psychiatry, Bucharest, Romania; ^4^Faculty of Medicine, Titu Maiorescu University, Bucharest, Romania; ^5^Colentina Clinical Hospital, Bucharest, Romania

**Keywords:** autism spectrum disorder, immune-related genes, cytokine, neuroinflammation, epigenetic factors

## Abstract

Autism spectrum disorder (ASD) is a neurodevelopmental condition characterized by communication and social interaction deficits, and by restricted interests and stereotyped, repetitive behavior patterns. ASD has a strong genetic component and a complex architecture characterized by the interplay of rare and common genetic variants. Recently, increasing evidence suggest a significant contribution of immune system dysregulation in ASD. The present paper reviews the latest updates regarding the altered immune landscape of this complex disorder highlighting areas with potential for biomarkers discovery as well as personalization of therapeutic approaches. Cross-talk between the central nervous system and immune system has long been envisaged and recent evidence brings insights into the pathways connecting the brain to the immune system. Disturbance of cytokine levels plays an important role in the establishment of a neuroinflammatory milieu in ASD. Several other immune molecules involved in antigen presentation and inflammatory cellular phenotypes are also at play in ASD. Maternal immune activation, the presence of brain-reactive antibodies and autoimmunity are other potential prenatal and postnatal contributors to ASD pathophysiology. The molecular players involved in oxidative-stress response and mitochondrial system function, are discussed as contributors to the pro-inflammatory pattern. The gastrointestinal inflammation pathways proposed to play a role in ASD are also discussed. Moreover, the body of evidence regarding some of the genetic factors linked to the immune system dysregulation is reviewed and discussed. Last, but not least, the epigenetic traits and their interactions with the immune system are reviewed as an expanding field in ASD research. Understanding the immune-mediated pathways that influence brain development and function, metabolism, and intestinal homeostasis, may lead to the identification of robust diagnostic or predictive biomarkers for ASD individuals. Thus, novel therapeutic approaches could be developed, ultimately aiming to improve their quality of life.

## Introduction

Autism spectrum disorder (ASD) is a complex neurodevelopmental condition with multifactorial etiology. The clinical picture is characterized by social interaction and communication deficits, as well as by restricted interests and stereotyped and repetitive behavior patterns (1). ASD prevalence has increased in recent decades, with current estimates of at least 1% of all children (2, 3). ASD is primarily a clinical diagnosis based on Diagnostic And Statistical Manual Of Mental Disorders, Fifth Edition (DSM 5) criteria. Depending on the degree of dependence on the entourage in the daily life, there are three levels of ASD severity: level 1 (requiring support), level 2 (requiring substantial support), and level 3 (requiring very substantial support) (1). ASD is characterized by an important clinical heterogeneity, related primarily to the severity of its specific features. Secondly, the heterogeneity lies in the association of other neurologic and psychiatric conditions such as epilepsy, motor abnormalities, intellectual disability, attention deficit hyperactivity disorder, sleep disorders, anxiety, and depression (4–7). In addition, a vulnerability to diverse medical conditions, such as gastrointestinal diseases, allergies, infectious disorders, immune dysfunctions, autoimmune conditions, and less common, congenital cardiac anomalies and hearing impairment, was observed in ASD populations (7, 8). Autism is currently considered a spectrum of deficits, across which a quantitative variation of behavioral and cognitive impairments is observed (9). In addition to the neurobehavioral phenotype, multiple systems and organs are involved, recent research suggesting that alteration of pleiotropic genes (i.e., a gene that independently influence several distinct phenotypic traits) and disruption of essential molecular mechanisms might underlie these various comorbid conditions (8).

ASD has a strong genetic component and a complex architecture characterized by an interplay of rare and common genetic variation (9–13). Various rare genetic variants with major individual phenotypic effect were discovered and proved to have a substantial contribution to ASD individual liability. However, each of these anomalies are extremely rare, accounting for <1% of ASD individuals (14–17). Common genetic variation, on the contrary, is estimated to collectively account as a major contributor to ASD liability, while individually has a small effect size (10, 12). The sustained efforts to decipher ASD genetics led to identification of many genes linked to ASD and neurodevelopmental disorders (NDDs). Autism resources such as SFARI Genes (https://gene.sfari.org/database/human-gene/, accessed August 5th 2022), includes 1,075 genes to date. One hundred and two genes were found to be strongly associated with ASD and NDDs risk in one of the largest whole exome sequencing studies (18). Immune response featured as one of the main biological pathways in which these genes were involved, beside neuronal communication, gene expression regulation, and cytoskeleton organization (18).

The extreme genetic heterogeneity of ASD is considered explanatory for the high variability of clinical presentations. However, there is a growing appreciation of the fact that the wide spectrum of genetic defects seems to disrupt common molecular functions and pathways (9, 19, 20).

Besides genetic factors and early lesions or immaturity of the brain, environmental factors may play a role in ASD, by themselves or in combination with the other risk factors. Air pollution during pregnancy, or in the first year of life (21, 22), as well as exposure during pregnancy to heavy metals, such as arsenic, cadmium, lead (23, 24), organic toxicants (25), or pesticides (26) were proposed as risk factors for ASD in offspring.

Nutritional deficiencies during pregnancy, such as low levels of Vitamin D (27) further discussed in this paper, were reported in various studies as factors that may contribute to an increase risk of ASD in offspring (27). In addition, gestational diabetes mellitus and hypertensive disorder of pregnancy may be associated with a risk of ASD in offspring, however further research is warranted to validate these findings (28, 29).

Genetic alterations in interaction with environmental risk factors converge toward the immune system (30). The genetic, epidemiologic, and immunologic studies bring new insights that can facilitate patient stratification, clinical management, and discovery of new therapeutic approaches.

The present paper reviews the updates regarding immune dysregulation, immune-related risk genes/pathways in ASD, highlighting some promising areas for potential biomarkers and therapeutic targets discovery.

## Immune system involvement in ASD

As the immune system is intricately linked to all systems and organs, it plays an important role in maintaining the entire body homeostasis. Crosstalk between the central nervous system and immune system has been hypothesized previously; recent evidence emerging from various studies of neuroanatomy, neuroendocrinology and cell biology began to elucidate the pathways connecting the brain to immune system (31). In this regard, maternal immune activation appearing in the first 3 months of fetal development has been suggested to be involved in the disruption of normal neurodevelopment (32). Numerous ASD gene expression studies pointed toward an immune system deregulation (33–35). Among the significantly upregulated genes in postmortem ASD brain tissues, many were identified to be involved in immune and inflammatory response and other immune regulatory processes (33, 34). Epigenomic players, such as microRNAs (miRNAs), were also shown to be dysregulated in ASD individuals, in blood and brain tissue (36, 37). These miRNAs target ASD genes and regulate, in correlation with transcription factors, complex metabolic and immune components (37). Additionally, genome-wide association studies brought new data on autistic-like traits or ASD that were associated with common variation affecting immune pathways (38, 39).

Dysregulation of both the innate and adaptive immune responses appear to be involved in ASD (40–42). The innate immune cells (e.g., monocytes, macrophages, and microglia) as well as immune molecules associated with innate immune responses were found to be altered in ASD individuals (40, 43–46). Elevated levels of cytokines, such as interleukin (IL)-1 β, IL-6, and tumor necrosis factor alpha (TNF- α), were detected in the plasma or postmortem brain samples of individuals with ASDs (40, 47–49). Besides monocytes, microglia have been the focus of intense investigation in ASD research. Microglia acts as an essential player in neural circuits formation; microglia activation, by up-regulated IL-1β, IL-6, IL-17, IL-18, IL-33, and TNF-α, has been proposed as a potential contributor to ASD pathogenesis (30, 34, 48, 50, 51). Adaptive immune responses are mediated by B and T lymphocytes (B-cells and T-cells) as a response to antigen exposure. B-cells contribute to the immune dysregulation in ASD, recent studies revealing high levels of pro-inflammatory cytokines (IL-6 and TNF-α) and low levels of anti-inflammatory cytokine - IL-10 in peripheral blood lymphocytes of ASD children (52). The analysis of T helper cells (Th cells) compartment and NK cell signaling pathways, placed at the interface between innate and adaptive immune responses, provided further evidence supporting the pro-inflammatory ASD environment (40, 42, 53, 54).

Systemic and central nervous system (CNS) inflammatory processes, through various immune cell compartments and specific cytokine networks, lead to a marked neuroinflammation suggested to contribute to ASD pathophysiology (50, 55–57).

### Cytokine dysregulation in ASD

Cytokines are intercellular signaling molecules, responsible for immune regulation and inflammatory responses; selected cytokines panels proved useful biomarkers when studying inflammation patterns (58–61). Cytokines are strictly linked to the immune response, although they can influence a wide range of different processes. Common and rare variants in cytokine genes, other regulatory genes or enhancer elements have been described in association with variation of cytokine levels among healthy individuals (62). Moreover, genetic variation regulating cytokine gene expression was associated with susceptibility to various immune-mediated and complex disorders (63, 64). The role of immune-related genes in ASD has been the focus of many recent studies. Among all known cytokines, IL-1 stands out as a molecule involved in many neuronal physiological pathways. IL1-β modulates neural plasticity and, historical examples show that IL1-β is necessary for long-term potentiation maintenance of hippocampal CA1 region (65). Animal model studies using specific antagonists, such as IL-1RA, showed that low levels of IL1-β are essential for normal synaptic plasticity (66), while abnormally elevated levels or depleted IL-1 lead to memory impairments (67, 68). Proinflammatory cytokines, such as IL-1, IL-6, TNF-α, and many others, have been reported to be dysregulated in various neuropsychiatric disorders (69, 70). Various members of IL-1 family, as main inflammatory cytokines, were investigated in different cohorts of ASD children. IL-1β triggers an inflammatory response through lymphocyte and macrophage activation. A chronic inflammatory state is sustained by increased tissue infiltration of inflammatory cells mediated by IL-1β (71). Another pro-inflammatory cytokine, IL-6 is an important immune factor involved in brain development; IL-6 impairs neurons' cellular adhesion and migration, as well as synapse development (72, 73). TNF-α is essential to inflammation regulation, especially inflammatory cytokine production (74); the impairment of TNF-α synthesis has long been suggested as relevant for immune disorders and complex diseases pathogenesis (75).

Analysis of peripheral cytokine messenger RNA (mRNA) expression has shown that *TNF-*α and *IL-6* were found statistically significant up-regulated in the blood (76) and in B cells of ASD subjects as compared to controls (52). Another study confirming the cytokine inflammatory status in ASD has shown that mRNA expression for *IL-1*β*, IL-4, IFN-*γ*, IL-9*, Janus Kinase 1 *(JAK1)*, and Signal transducer and activator of transcription 5 *(STAT5)*, in peripheral blood mononucleate cells (PBMC), was found significantly elevated in ASD individuals compared to controls (77). *IL-31*, coding another cytokine involved in chronic inflammation (78) was found to be increased in ASD. Pro-inflammatory cytokines production in stimulated monocytic cells from peripheral blood was investigated by Enstrom et al. in ASD (45). After stimulation with toll-like receptors ligands, the monocytes had a significant increase in IL-1 β, IL-6, and TNF-α secretion in ASD children compared to typically developing age-matched controls. Ashwood et al. found increased IL-1β, IL-6, IL-8, IL-12p40 plasma levels in children with ASD; the increased cytokine levels seemed to be associated with more severe symptoms of core ASD domains (40). Another study has shown that ASD children had increased levels of TNF-α protein and decreased expression of TNF and HNRNPL-related immunoregulatory long non-coding RNA (*THRIL*) gene. *THRIL* was shown to negatively regulate TNF-α expression in macrophages, thus this regulatory pathway could be disturbed in ASD individuals (79). The elevated peripheral proinflammatory cytokines landscape showed a potential correlation with ASD comorbidities, namely epilepsy; higher levels of IL-12p40 were detected in ASD individuals having a positive seizure history compared with ASD and no epilepsy (54).

IL-16, a chemoattractant that modulates T cell activation was studied in ASD's PBMCs and several dysregulations were found. Thus CD4+IL-16+, CD8+IL-16+, CD14+IL-16+, CCR3+IL-16+, and CXCR7+IL-16+ cells were found increased in ASD with a concomitantly increased expression of IL-1β+IL-16+, IL-6+IL-16+, and TNF-α+IL-16+. All these results qualify the chemoattractant IL-16 as another driver of immune alteration (80).

In addition, several meta-analyses found significantly increased peripheral pro-inflammatory cytokine levels, such as IL-1β, IL-6, and IFN-γ and decreased levels of transforming growth factor (TGF)-1β as an anti-inflammatory cytokine, in ASD individuals compared to controls (69, 81, 82).

Cytokine studies performed on blood spots from neonatal cohorts allowed the assessment of circulating levels prior to an ASD diagnosis. Krakowiak et al. showed that IL-1β and IL-4 circulatory levels detected at birth are independently associated with ASD, the clinical diagnosis being established from 2 to 5 years of age. A correlation with ASD severity was also observed, IL-4 being associated with severe forms of ASD (according to Autism Diagnostic Observation Schedule score) and IL-1β with mild ASD. The increased expression of IL-1β and IL-4 indicate prenatal immune abnormalities, and thus support a potential contribution to ASD pathogenesis (83). In another study, higher concentrations of IL-6 and IL-8 were found in neonatal blood spots from individuals who later developed ASD compared to the general population. In addition, significantly increased eotaxin-1, IFN-γ, and IL-12p70 levels were found when comparing ASD with children with developmental delay (84).

Concordant results were obtained for adult psychiatric disorders where circulatory cytokine levels were significantly increased (IL1-RA, IL-18, TNF, IL-6, and C-Reactive Protein). Over-expression of genes coding inflammatory molecules was also observed to be positively correlated with disease severity (85).

Adaptive immune responses are triggered by exposure to antigens and are generally mediated by Th cells. Th cells can be categorized, depending on the cytokines produced and their functional consequences, into pro-inflammatory Th1 or anti-inflammatory Th2. Although various cytokine studies in ASD children reported increased levels of either Th1 (40, 45, 86, 87) or Th2 cytokines (88), the evidence converge toward a pro-inflammatory ASD environment (40, 53, 54).

The immune dysfunction observed in ASD individuals is also supported by post-mortem brain tissue and cerebrospinal fluid (CSF) studies. Vargas et al. showed evidence for active inflammatory processes in the cerebral cortex and cerebellum of ASD patients, supported by elevated levels of cytokines such as chemoattractant protein (MCP)−1 and IL-6 (48). Another study showed the presence of elevated levels of IL-6, IL-8, TNF-α, granulocyte-macrophage colony-stimulating factor (GM-CSF), and IFN-γ, with no significant differences for IL-4, IL-5, and IL-10 between port-mortem brain samples from ASD and control groups, thus pointing toward Th1 pathway activation and subsequently increased adaptive response (49). Increased levels of IL-6 were found by Wei et al. in the cerebellum of post-mortem tissue samples from ASD individuals and were interpreted as potential contributors to the alteration of the balance between excitatory and inhibitory circuits (89).

CSF also proved valuable for immune-related detection of biomarkers in ASD. Smedler et al. analyzed over 200 proteins from CSF and serum collected from twins with various neurodevelopmental conditions including ASD, in a study aimed to detect ASD markers. The study showed that autistic behavior and ASD were associated with serum B-cell activating factor (BAFF) and Cystatin B (CSTB) (90). BAFF is a cytokine belonging to the TNF ligand family, known to be involved in autoimmune disorders (91), as well as in psychiatric disorders, such as schizophrenia and bipolar disorder (92). In an animal model of ASD, it was shown that TNF superfamily member 13b gene (*Tnfsf13b*), encoding BAFF, is up-regulated in the prefrontal cortex; moreover, the study proposed *Tnfsf13b* among the immune genes that may play a role in social behavior regulation (93).

Although there are many studies supporting a proinflammatory component of ASD and immune dysregulation, some limitations of these studies must be taken into account. As cytokine concentrations and ratio can fluctuate, standardization is required with regard to the analysis type, sample source and the molecular player examined (gene, mRNA, protein and so on) (94).

**Figure 1:**
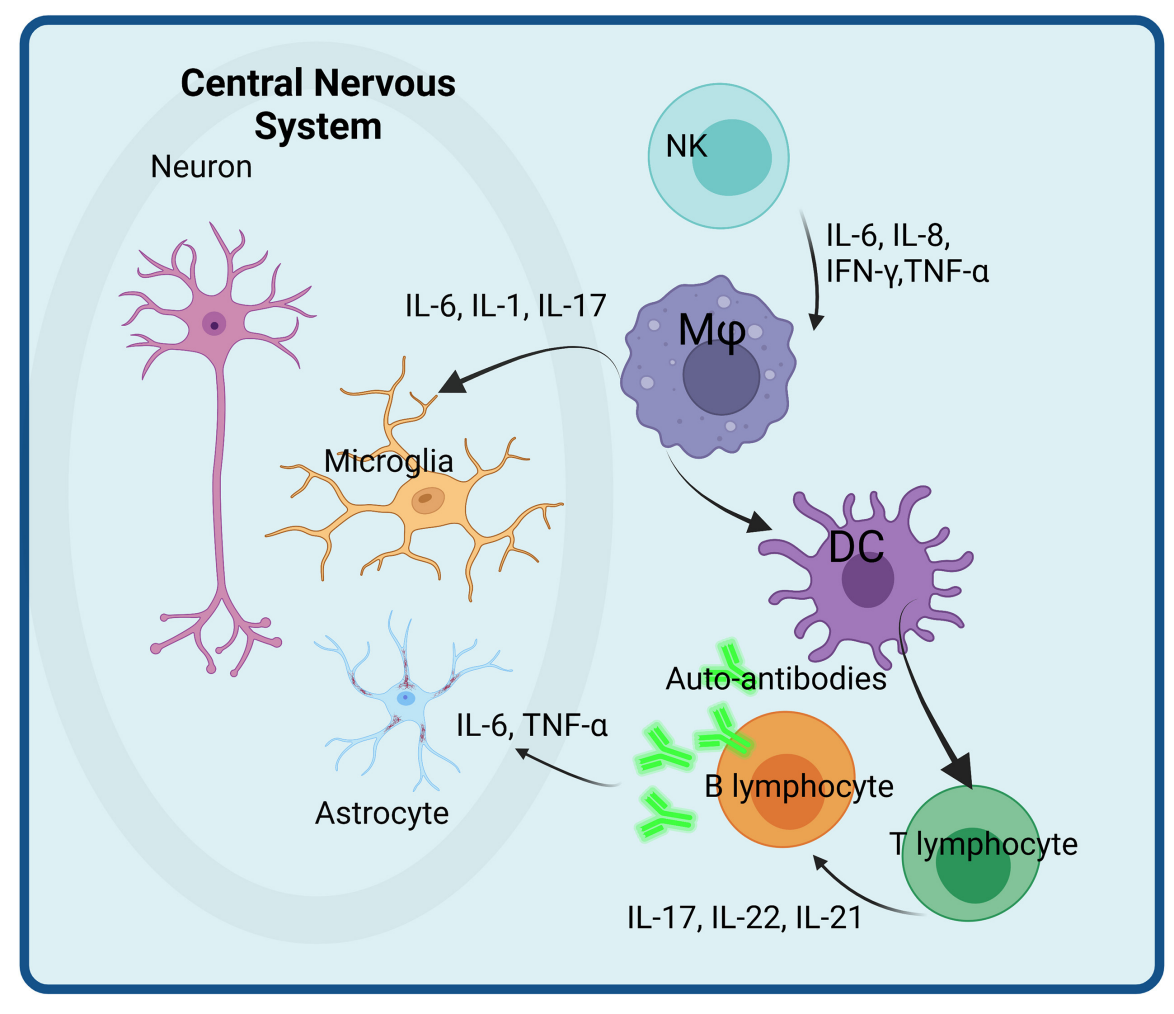
Immune cells and their secreted immune-related molecules with potential roles in neuro-inflammation in ASD. Activated NK cells secrete pro-inflammatory cytokines (IL-6, IL-8, IFN, and TNF) that activate macrophages (Mφ) with enhanced secretion of IL-6, IL-1, and IL-17 that impacts directly the microglial populations. Activated dendritic cells (DC) play a role in T and B lymphocytes activation, that enhance the pro-inflammatory cytokine secretion as well as the differentiation of B cells in plasma cells. The auto-antibodies thus produced further attack astrocytes. Created with BioRender.com.

An overview of the main immune cells and their secreted cytokines involved in neuro-inflammation in ASD are presented in Figure 1.

### HLA and immunoglobulins dysregulation in ASD

The major histocompatibility complex (MHC) region is a complex genomic system localized on chromosome 6p21.3p22.1. It encompasses the human leukocyte antigen (HLA) gene cluster, that has important biological roles in immune system activity, as well as in neurodevelopment and neuroplasticity (41, 95–98). HLA cluster is one of the most polymorphic regions in the human genome and includes three distinct functional classes annotated from I to III. Considering the variety of functions of HLA genes in immune regulation and nervous system development and homeostasis, numerous studies suggested a connection between HLA alleles/haplotypes and psychiatric disorders (35, 99, 100). Several association studies revealed specific HLA alleles related to ASD, such as *HLA-DRB1* and *HLA-A2* (101–103). However, the analysis of the complex MHC region in large autism genome-wide association studies (GWAS) was hindered by the complexity and haplotype diversity of this region, thus precluding further association of HLA alleles and autism (12, 100).

Tissue expression levels of the HLA-DR alpha (HLA-DRA), MHC class II cell surface receptor, were significantly reduced in the gray matter of post-mortem ASD brain samples compared to control samples. In addition, the expression of the Mannose Receptor C-Type 1 (*MRC1*), an anti-inflammatory gene, was found significantly increased in the white matter of ASD individuals. MRC1+ cells are involved in the neuroinflammatory cellular processes, namely in the removal of apoptotic/necrotic cells (47).

The pro-inflammatory microglia phenotype is characterized by HLA-DR and CD68 expression. HLA-DR is mainly involved in antigen presentation required for T-cell function, while CD68 is mainly associated with phagocytosis functions (47). The alteration of their expression in the post mortem ASD brain samples differs with regard to the studied cerebral area (47, 48). MRC1 activation induces IL-10 secretion, while IL-10 induces MRC1 expression in cultured macrophages (104). The inflammatory alterations reported by Sciara et al. within the brain tissue reside in the increased vasculature areas and may lead to altered myelination and, ultimately, may contribute to the complex ASD phenotype (47).

In another study evaluating post-mortem brain tissue in ASD, DiStasio et al. showed that the perivascular lymphocytic cuffs display increased numbers of lymphocytes in over 60% of the investigated ASD samples. Total T lymphocytes predominate over B lymphocytes and cytotoxic T CD8+ over helper CD4+ T lymphocytes. These perivascular lymphocytic infiltrates are associated with astrocyte blebs irrespective of the diagnostic age. The authors suggest that the astrocytic blebs are the result of cytotoxic T-lymphocytes. Taking into account the association of ASD with specific HLA alleles and MHC molecules, the authors suggest that cytotoxic T CD8+ lymphocytes in ASD target MHC-expressing astrocytes (105). The damaged astrocyte, damage performed directly or cytokine-mediated, cannot offer metabolic support to axons, potentially impairing axon function (106). This assertion sustains firstly the direct immune involvement in the neuronal physiology and secondly depicts the involvement of other related pathways, like the metabolic ones. An interesting parallel with other autoimmune diseases, has shown that in Type 1 Diabetes, specific HLA alleles are associated with CD8+ T-lymphocyte autoreactivity and islet autoimmunity promoting pancreatic islet cell destruction (107, 108). Hence, MHC alleles specific to ASD were reported several years ago (103). Another hypothesis proposes that astrocyte/immune cells related markers detected in CSF or serum can represent future biomarkers in ASD (105).

Immunoglobulins (Ig) represent another category of immune-related molecules that have been recently gaining interest in ASD research. A review study published in 2012 focused on the maternal-fetal transfer of brain-reactive antibodies and its impact on the risk of developing ASD suggesting that these antibodies may play a role in the behavioral outcome (109). Also, cerebellar-specific autoantibodies were found in children with ASD, correlated with lower adaptive and cognitive function; however, the study could not determine a clear pathogenic significance of this phenomenon (110). These antibodies cross the placenta, recognize self-proteins and can hinder fetal development, as blood brain barrier is still not completely formed (111). Therefore, maternal autoantibodies may be proposed as markers for ASD diagnosis (35, 112). Autoantibodies toward folate receptor alpha (FRα) were also found in ASD children, autoantibodies that are involved in autoimmune diseases development and oxidative stress (113). These autoantibodies hinder folate passage across the blood-brain barrier to the brain. The mothers of ASD children can have these types of autoantibodies leading to an impairment of folate passage across the placenta. Another interesting area is the rather complex folate-dependent one-carbon metabolism including the methylation cycle, the trans-sulfuration pathway and the folate cycle (114). These metabolic pathways are linked to the DNA synthesis/repair, DNA, RNA and proteins methylation, oxidative stress, cellular proliferation/apoptosis, and many other processes (114) linking other complex pathways that can relate to the pathophysiology of ASD (115). Moreover, FRα autoantibodies that can impair folate transport and oxidative biomarkers can lead to the discovery of new therapeutic strategies (113).

Another interesting Ig molecule was recently associated with ASD. IgA concentration and specificities are associated with multiple factors (e.g., subject age, gut microbiota composition, T cell abundance) (116). Hence, IgA was reported as significantly elevated in the gut of ASD children (117). Virulence factor-related gut microbiota (VFGM) genes were found positively correlated to the IgA levels of ASD children; a specific VFGM gene configuration was associated with ASD. VFGM genes detected in ASD were found to be more diverse as compared to typically developing children (118). Group B streptococcus (GBS) genes represented the most prominent VFGM group in ASD. In ASD animal models it was shown that maternal GBS can induce autistic-like litter (119). Wang et al. have shown that three bacterial lipopolysaccharide (LPS) genes (e.g., *kfiC, Cj1137c, wlaN*) were found significantly enriched in gut microbiota of ASD children, positively correlating with gut IgA and VFGM gene diversity (118). This study confirmed the findings of an earlier experimental animal study (120). Therefore, recent studies pinpoint that there are clear links within the immune-gut-brain axis in ASD (121, 122), as further elaborated in the paper. Moreover, the regulation of intestinal microbiota of pregnant female mice was shown to prevent ASD-like behaviors in their offspring corroborated with a normalization of pro-inflammatory cytokines IL-6 and IL-17a (123). Therefore, dysregulated intestinal microbiota and its potential role in sustaining the inflammation in ASD is a topic to be developed in the near future (118).

### Immune-related genes and pathways

In immune systems cells, cystatins are involved in antigen processing and presentation, in phagocytosis, cytokines modulation, and nitric oxide expression (124). Concomitantly, cystatins are involved in neurogenesis processes and potentially in synaptic plasticity, as showed in human studies and animal models (125, 126). Cystatin B (CSTB) serum level was strongly associated with autistic features in a recent twin study on ASD and other neurodevelopmental disorders (90). Moreover, Unverricht-Lundborg disease is a rare recessive myoclonic epilepsy caused by pathogenic variants in *CSTB* gene (omim.org/entry/254800, accessed on April 20, 2022). Although the typical phenotype does not include autistic features, a recent report of three patients with Unverricht-Lundborg disease brought into attention ASD as part of the clinical picture (127). Taking into account the potential roles of *CSTB* in the nervous system, future studies are needed to establish if ASD is associated with certain genetic defects in the *CSTB* gene.

Several other immune-related genes involved in immunologic disorders have also been investigated in association with ASD. One example is the adenosine deaminase (*ADA*) gene encoding the adenosine deaminase which plays an important role in purine metabolism. Adenosine deaminase has an important role in immune system function. When mutated it induces a severe combined human immune deficiency (SCID), profound lymphopenia on all classes and a dysfunctional purine metabolism (128). Certain *ADA* alleles were reported as associated with ASD in earlier studies. A significantly increased frequency of *ADA* Asp8Asn polymorphism (*ADA2* allele) was reported by two independent case-control studies of individuals with autism of Italian descent (129, 130). This variant has been associated with a low-activity of the ADA enzyme (129). However, no significant increase in the frequency of the ADA2 allele was observed in a case-control study of North America ASD families, the authors suggesting the existence of a potential risk haplotype in the Italian ASD population (131). As other studies reported neurological and behavioral problems in patients with ADA deficiency and SCID, but not autism (132, 133), further studies are warranted in order to dissect the *ADA* gene role in ASD.

Fibroblast growth factor (FGF) family is involved in complex regeneration processes and initiates the inflammatory events (134), having roles in brain development, regulating cortical size and connectivity as well (135). By using whole exome sequencing to investigate Saudi families with ASD members, Al-Mubarak et al. detected a rare variant of *FGF5* among other risk genes with roles in brain development and function (136). Several lines of evidence suggested that dysregulation of FGF signaling may contribute to ASD pathophysiology (137–139). Specifically, the macrocephaly observed in early childhood in ASD which is attributed to increased head growth mainly in the first 2 years of life, is hypothesized to be, at least in some children, caused by alterations in *FGF* genes expression (135). Similarly, the lipid and protein phosphatase and tensin homolog (*PTEN*) regulates the physiology of many immune cells as well as embryonic stem cells proliferation, including the neurogenic ones (135, 140). The signaling downstream from cytokine and T- and B-cell receptors, integrins, and growth factor receptors depends on PTEN activity. Therefore, mutations in *PTEN* gene have tremendous effect, like dysfunction of the immune system, autoimmunity, and lymphoid hyperplasia (141). Germline mutations in *PTEN* were initially reported in individuals with autosomal dominant forms of familial tumor predisposition syndromes (142, 143), currently termed as PTEN hamartoma tumor syndrome; some of these presented neurodevelopmental problems, including ASD (144). Several studies reported heterozygous germline *PTEN* mutations in individuals with macrocephaly, developmental delay and ASD, leading to delineation of a new syndrome, Macrocephaly/autism syndrome (MIM605309) (145, 146). Studies on animal models showed that *PTEN* selective deletion in cerebral cortex and hippocampus neurons leads to macrocephaly and behavioral abnormalities (147). The individuals with *PTEN* variants display a various spectrum of immune dysfunction. This varies from asymptomatic lymphopenia to different forms of lymphoid hyperplasia, such as hyperplasia of the adenoids and/or tonsils leading to recurrent upper respiratory tract infections, gastrointestinal polyps with follicular lymphoid hyperplasia, adenoid lymphoid hyperplasia, and thymus hyperplasia (148–150).

In the last years, numerous studies revealed an overlap between autism and cancer genes with many common genes involved in major cell-signaling pathways and metabolic processes dysregulation (151). Among these, the genes involved in PI3K-Akt-mTOR signaling axis, such as *PTEN, NF1* (neurofibromin 1)*, TSC1*(TSC complex subunit 1)*, TSC2* (TSC complex subunit 2), were associated with inherited risk for both cancer and ASD (152, 153).

Alternative splicing and co-expression analyses of total RNA from PBMC isolated from ASD twins and their parents has shown that zinc finger protein 322 (*ZNF322)* and nuclear receptor subfamily 4 group A member 1 (*NR4A1)* display differentially alternative splicing (154). ZNF322 is a member of the zinc-finger transcription factor family with a putative role in regulation of the ubiquitous MAPK signaling pathways (155) while NR4A1 is a key general regulator in the induction of T cell dysfunction (156). Since the genes coding these molecules seem to play crucial roles in their networks, further studies for their testing and validation as biomarkers for ASD are needed.

Another molecule linked to the immune system function is vitamin D. Besides the roles played in calcium homeostasis, vitamin D has important immune functions (157). Low levels of vitamin D were reported in association with increased levels of proinflammatory cytokines in various disorders such as cancer and psychiatric conditions (158–162). Specifically, vitamin D metabolites have a regulatory repressive effect on IL-8 promoter activation, through vitamin D receptor (VDR) stimulation (163). Animal model studies and human epidemiological studies also suggested that vitamin D deficiency has an important impact on nervous system development (164–166). The assessment of vitamin D deficiency during pregnancy in two population-based cohorts from the Netherlands (165) and Sweden (166) showed association with a greater risk of ASD occurrence in offspring. As vitamin D acts upon binding to vitamin D receptor (VDR), common variants in *VDR* gene (e.g., FokI, BsmI, ApaI, and TaqI polymorphisms) were investigated for their functional consequences on the Vitamin D-VDR complex. These polymorphisms were also investigated in association with various disorders such as immune disorders, cancer, and neuropsychiatric disorders, including autism (167–170). Although some positive associations were found, conflicting results were also generated drawing the attention toward factors such as methodological and cohort differences between the associations studies, as well as environmental effect upon genes and gene-to-gene interactions. A more recent genotyping study of the above-mentioned polymorphism in ASD children, their parents, healthy siblings and controls showed that FokI polymorphism was robustly correlated with ASD; in addition, the frequency of FokI polymorphism in mothers of ASD children revealed an increased risk of having a child with ASD. Authors point out that FokI (T) minor allele codes for a less active protein in comparison to the FokI (C) allele. Therefore, a reduced biological activity of VDR-vitamin D complex is envisaged which may explain the sustained inflammation in pregnant mothers and ASD children with FokI (TT) genotype (171).

### Mitochondrial biology dysfunctions and oxidative stress in ASD

The imbalance between production and removal of reactive oxygen species (ROS) generating oxidative stress has recently gained attention in autism research (172–175). ROS are physiologically produced to kill pathogens or as metabolic intermediates. ROS are generated from electron transport chains, drug metabolism, from exposure to chemicals, pollutants, and/or upon radiation. Nicotinamide adenine dinucleotide phosphate oxidase (NOX) isoforms generate endogenous ROS, and are localized to various cellular membranes being involved in acute and chronic brain diseases (176). Monocyte—macrophage lineage and neutrophils are the main immune cells that generate ROS. ROS constitute the first line of defense against pathogens, but these species also regulate the activity of innate and adaptive immunity. As a primary or secondary signal molecule, ROS are involved in various pro-inflammatory mechanisms (177) including neuro-inflammation (178). B lymphocytes from ASD individuals showed increased gene expression of toll like receptor 4 (*TLR4*) and nicotinamide adenine dinucleotide phosphate oxidase 2 (NADPH oxidase2—*NOX2*) (179). At the same time, T cells displaying the similar feature and contributing to the oxidative stress governed by innate cells (180) (Table 1).

**Table 1:** Reactive oxygen species and anti-oxidants generated by immune cells in ASD.

**Oxidative stress**					
**Oxygen species**	**Enzyme**	**Innate immune cells**	**Adaptive immune cell**	**ASD**	**References**
ROS: superoxide (O2–), hydroxyl (OH), hydrogen peroxide (H2O2), singlet oxygen (1O2)	Nicotinamide adenine dinucleotide phosphate (NADPH) oxidase	Monocyte, macrophage, neutrophils	B lymphocytes increased NADPH oxidase	Increased generation	(176–178)
			T lymphocytes increased NADPH oxidase		(180)
Hypochlorous acid (HOCl)	Myeloperoxidase (MPO)	Granulocytes	–	Increased generation	(182)
Nitric oxide (NO)	Inducible nitric oxide synthase (iNOS)	Monocyte, macrophage, dendritic cells, neutrophils	–	NO is neurotoxic	(174, 180)
**Antioxidant mechanisms**					
**Enzyme**	**Action**	**Cell source**		**ASD**	**References**
Superoxide dismutase (SOD)	Conversion of superoxide to H2O2	All cells		Increased levels are found in neutrophils and monocytes	(113, 187, 190)
Glutathione peroxidase (GSHPx)	Inactivation of free peroxides in cells	All cells		Decreased activity in neutrophils and monocytes	(190)
Glutathione reductase (GR)	Provides reduced glutathione to control ROS	All cells		Decreased activity in neutrophils; unchanged activity in monocytes	(190)

Another enzyme, myeloperoxidase (MPO) is primarily located in immune cells and plays an important role in the immune system, which produces some ROS, particularly hypochlorous acid (HOCl) having the purpose to kill invading pathogens (181). A recent study showed that serum MPO was found significantly higher in ASD compared to control subjects (182). Another important species, nitric oxide (NO) is generated by NO synthases (NOSs) involved in physiological and pathological conditions (183, 184). Interestingly, low concentrations of NO generated by neuronal NOS or endothelial NOS have a physiological neuroprotective function and are involved in the signaling pathway, while higher concentrations of NO synthesized by inducible NOS (iNOS) are neurotoxic (183, 184). iNOS was found in various cell types of the immune system, such as macrophages, dendritic cells, neutrophils, to non-immune cells like epithelial cells from the gut and lung mucosa, smooth muscle cells, and stromal cells of secondary lymphoid organs (178). This was linked to ASD (185) and may be involved in damaging the DNA as well as the enzymatic apparatus that regulates the neurotransmitters (174, 180). Multiple dysregulated molecular players were identified by various studies focused on the oxidative stress in ASD children.

Increased gene expression of *IL-6* and of *HSP70i* (a stress protein) and increased plasma levels of peroxiredoxin (2 and 5) were found by Abruzzo et al. in ASD children compared to controls (186). Peroxiredoxin is a peroxide scavenger, molecular chaperone and contributor to modulation of the cytokine storm Assuming the role of stress proteins involvement in neuroinflammation, this opens avenues for potential ASD's new therapeutic approaches and for plasma peroxiredoxin as a possible biomarker/indicator of disease severity (186).

Significant increase of oxidative DNA damage was found in peripheral lymphocytes from ASD individuals, along with increased plasma ceruloplasmin and copper concentrations, thiol proteins, and superoxide dismutase (SOD) levels, while vitamin C and A levels had lower values in comparison to controls (113). SOD enzymes play critical roles in cell protection against ROS by converting the superoxide to hydrogen peroxide (H2O2) (187). Sequence polymorphisms in the genes encoding for SOD enzymes, namely *SOD1, SOD2*, and *SOD3* were investigated in several human disorders (187–189). *SOD1* is associated with familial amyotrophic lateral sclerosis (MIM **#** 105400), mutations in this gene being reported in ~20% of patients (https://www.omim.org/entry/105400, accessed on April 22, 2022). Two single nucleotide polymorphisms in *SOD1* (rs2234694 and rs36233090) were reported in correlation with an increased ASD risk. These variants are localized in non-coding regions of the gene and are predicted to have regulatory effects (187). It was recently shown that within harvested neutrophils and monocytes from ASD patients there is an increased SOD expression. However, this is associated with upregulated expression of nitrotyrosine (a marker of oxidant damage), proving the dysregulated antioxidant network (190). Similar results were obtained in T cells from ASD patients, namely an increased antioxidant potential coupled with an inflammatory pattern (191).

Another recent study explored the genetic polymorphisms of glutathione transferases (GSTs) in ASD (192), enzymes with multiple functions, such as inactivation of epoxides and hydroperoxides, molecules generated during oxidative stress. Moreover, GSTs are involved in the synthesis of important biomolecules such as prostaglandins, leukotrienes, and hormones (testosterone and progesterone) (193). Six GSTs gene subfamilies have been described (194), with several highly polymorphic members. Several polymorphisms which influence gene transcription and cause functional alterations at protein level were identified, such as homozygous deletions in glutathione S-transferase mu 1 *(GSTM1)*, glutathione S-transferase theta 1 *(GSTT1)* and single nucleotide polymorphism in glutathione S-transferase pi 1 (*GSTP1)*, glutathione S-transferase alpha 1 *(GSTA1)* genes (192). *GSTM1* null allele was identified as a potential risk factor of ASD in offspring of mothers receiving medication during pregnancy. This highlights the fact that oxidative stress-related genetic factors added to the environmental factors may contribute to ASD development (192).

Mitochondria play a myriad of physiological functions in metabolism (e.g., glucose oxidation, biosynthesis of fatty acid, amino acid and hormones), ROS signaling, cellular survival regulation including apoptosis and innate immunity (195). Recently reviewed by Chen et al. mitochondrial dysfunction and oxidative stress activate the innate immune system with deleterious roles in inflammatory-related (196) and various autoimmune diseases (197), cancer (198), neurological disorders (199), and diabetes (200). Bennuri et al. studied the consequences of prolonged ROS exposure in a lymphoblastoid cell line (LCL) model of ASD, mainly the adaptive changes that occur in mitochondria function. Gene expression changes were detected, with increased expression for *SOD2*, uncoupling protein 2 *(UCP2)*, and mammalian target of rapamycin kinase (*MTOR)* genes. Also, an increased expression of protein kinase AMP-activated catalytic subunit alpha 2 (*PRKAA2)* gene was observed with potential functional consequences on mTORC1 (201). All these genes are highly involved in mitochondrial respiration. Prolonged exposure to ROS induced changes in mitochondrial respiration. It was also suggested that mTORC1 pathway regulates mitochondrial activity in ASD in relation to S6 kinase beta-1 (S6K1) regulation. S6K1 pathway activated by mTORC1, can sustain mitochondrial response to stress. Therefore, in the context of chronic oxidative stress, normalizing mitochondrial functionality in ASD individuals can gain new therapeutic value (201). In a Chilean cohort of children with ASD a significant increase of the mitochondrial DNA levels was reported along with an increase in the protein oxidation (189). The study also proposed a screening of gene expression in a panel of genes relevant for mitochondria function, consisting of HIG1 Hypoxia Inducible Domain Family Member 2A *(HIGD2A)*, superoxide dismutase 2 (*SOD2)*, mitofusin 1 (*MFN1)*, mitofusin 2 (*MFN2)*, dynamin 1 like (*DRP1)*, fission mitochondrial 1 (*FIS1)*, and OPA1 mitochondrial dynamin like GTPase (*OPA1)*. However, the only gene expression change that reached statistical significance was the increased expression of *MFN2* (189). Mitochondria are organelles characterized by structural and molecular dynamics undergoing fusion and division (202). All the above-mentioned genes are involved in the fusion/division processes in mitochondria. Probably the increase in mitochondrial DNA levels identified in ASD children is a compensatory process aimed at maintaining the mitochondrial functions (189).

Oxidative stress, caused by ROS or reactive nitrogen species, may disrupt the homeostasis of many cells and tissues, leading to mitochondrial and metabolic dysfunctions, altered immune responses, and central and systemic inflammation (177). The full understanding of the role of oxidative stress in ASD is still a matter of intense research and may open future perspectives for a better understanding of ASDs.

Table 1 depicts oxidative stress and antioxidants in different immune cells (B cells, T cells, monocytes, neutrophils) relevant for ASD individuals.

**Figure 2:**
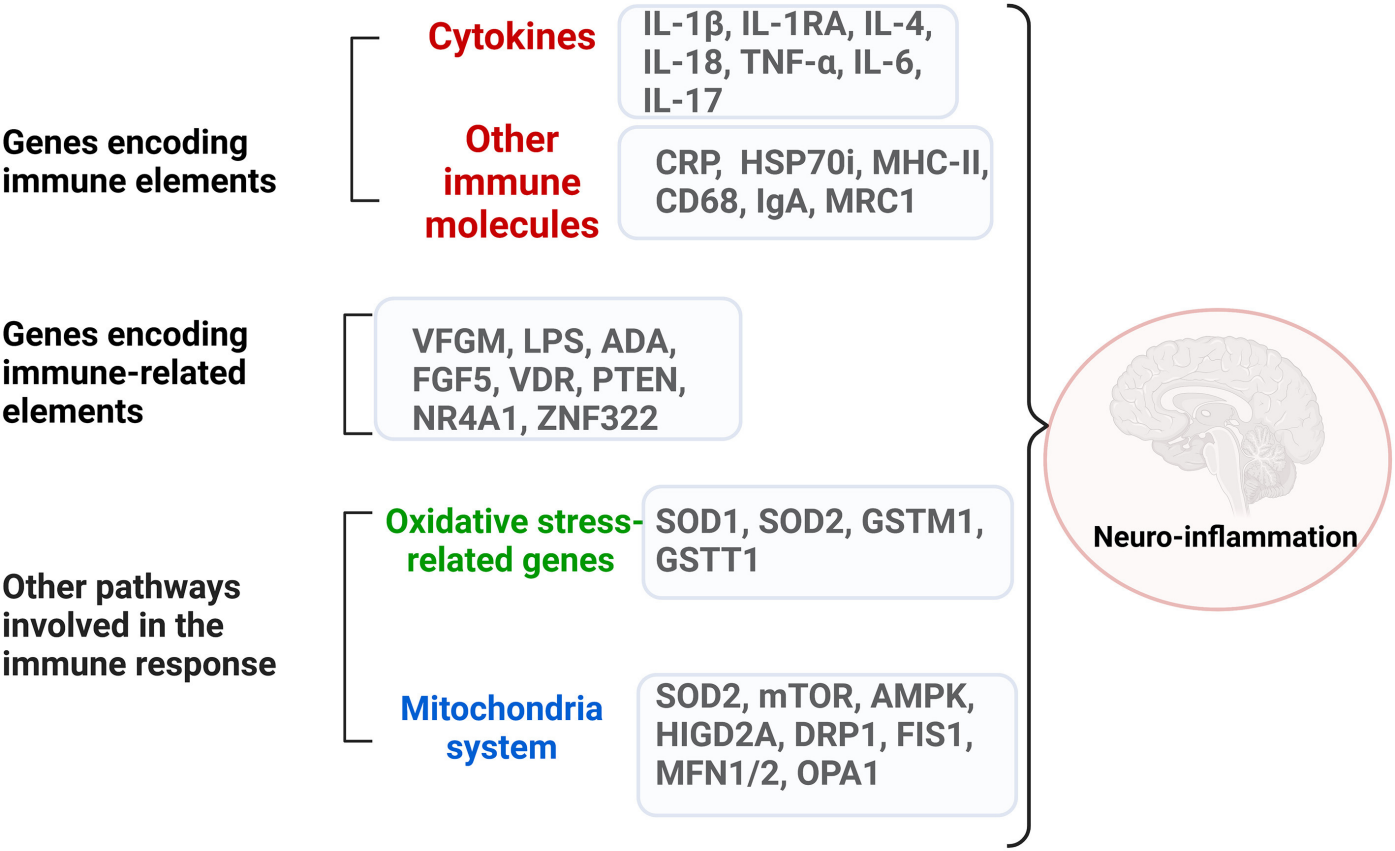
The complex network of dysregulated genes encoding immune system elements and immune-related ones in ASD. Neuroinflammation can be generated through dysregulated genes from three categories: genes that encode directly immune molecules, like cytokines and surface molecules on the immune cells; genes that encode elements involved in major immune pathways like growth factors, proliferation processes; genes that encode molecules involved in oxidative stress and mitochondria pathways. Created with BioRender.com.

The complex network of genes that encode immune systems elements and immune-related ones in ASD is briefly outlined in Figure 2 and summarized in Table 2.

**Table 2:** Summary of immune-related genes with dysregulated expression in various ASD studies.

**Gene name**	**Gene alteration detected in ASD studies (ref)**	**Protein function (https://www.uniprot.org/; https://www.ncbi.nlm.nih.gov/protein/)**
*NADPH* oxidase2—*NOX2*	Increased gene expression (179)	Major subunit of the phagocytic NADPH oxidase which generates superoxide
*HLA-DRB1*	Human Leukocyte Antigen (HLA)-DR4 polymorphic allele (103)	Part of HLA class II beta chain molecules. Presents antigens on antigen presenting cells (APC), guiding antigen-specific T-helper effector functions
*HLA-A*	HLA-A2 polymorphic allele (103)	Belongs to HLA class I molecules. Presents antigens on APC cells, guiding functions of antigen-specific cytotoxic CD8-positive T cells
Heat Shock 70 KDa Protein 1A—*HSP70I*	Increased gene expression (186)	Chaperone molecule involved in various cellular processes, with an essential role in the protein quality control system
Interferon-gamma IFN-γ—*IFNG*	Increased gene expression (52)	Belongs to type II interferon class molecules. Soluble cytokine which is produced by innate and adaptive immune system cells
Interleukin 1 Receptor Antagonist—*IL1R1*	Increased gene expression (85)	Belongs to interleukin 1 cytokine family. Inhibits the activities of IL-1A, IL-1B and modulates a variety of immune and inflammatory responses related with IL-1
Interleukin 6—*IL-6*	Increased gene expression (52, 76, 186)	Cytokine involved in various biological functions, with important role in immunity, tissue regeneration, and metabolism
Interleukin 9—*IL-9*	Increased gene expression (77)	Pleiotropic cytokine involved in immune response, regulates the function of various hematopoietic cells, stimulates cell proliferation, and prevents apoptosis. IL-9 stimulates a receptor complex (IL-9R/IL2RG) which leads to activation the JAK-STAT pathway
Interleukin 10—*IL-10*	Decreased gene expression (52)	Pleiotropic cytokine with immunosuppressive activity. Receptor complex ligation activates JAK/STAT signaling
Interleukin 16—*IL-16*	Increased gene expression (80)	Pleiotropic cytokine with chemoattractant function for a variety of CD4+ immune cells
Interleukin 17A—*IL-17A*	Increased gene expression (52)	Member of the IL-17 receptor family. Proinflammatory cytokine produced by activated T cells
Mannose receptor C-type 1—*MRC1*	Increased gene expression (47)	Type I membrane receptor involved in mediation of glycoproteins endocytosis by macrophages
Mammalian target of rapamycin—*MTOR*	Increased gene expression (201)	Serine/threonine protein kinase with central role in regulation of many fundamental cell processes: cellular metabolism, growth and survival in response to hormones, growth factors, nutrients, energy, and stress signals. mTOR constitute the catalytic subunit of two distinct protein complexes: mTORC1 which promotes translation initiation and controls protein synthesis, and mTORC2 which is a regulator of the actin cytoskeleton, and promotes cell survival and cell cycle progression
Neurofibromin 1—*NF1*	Rare sequence variants (https://gene.sfari.org/database/human-gene/, accessed August 5th 2022)	Multifunctional protein involved in several cell signaling pathways (Ras/MAPK, Akt/mTOR, cAMP/PKA) and regulates fundamental cellular processes, such as proliferation and migration, cytoskeletal dynamics, neurite outgrowth, dendritic-spine density, and dopamine levels
Protein kinase AMP-activated catalytic subunit alpha 2—*PRKAA2*	Increased gene expression (201)	Catalytic subunit of AMP-activated protein kinase (AMPK), an important energy-sensing enzyme with critical role in cellular energy status
Phosphatase and tensin homolog—*PTEN*	Rare sequence variants (https://gene.sfari.org/database/human-gene/, accessed August 5th 2022)	Enzyme with tumor suppressor function. Presents dual specificity for protein and lipid phosphatase. Regulates important cellular processes, such as proliferation, differentiation, growth, migration, death, apoptosis of the cells through the PI3K/AKT/mTOR signaling pathway
Superoxide dismutase 1—*SOD1*	Single nucleotide polymorphisms (187)	An antioxidant enzyme that metabolizes the free superoxide radicals in the body to molecular oxygen and hydrogen peroxide
Superoxide dismutase 2—*SOD2*	Increased gene expression (201)	Member of the iron/manganese superoxide dismutase family with important role in oxidative stress management
Toll like receptor 4—*TLR4*	Increased gene expression (179)	Member of the TLR family which plays an essential role in regulation of immune responses to infection
Tumor necrosis factor alpha—TNF *(TNF-α)*	Increased gene expression (52, 76)	Multifunctional proinflammatory cytokine that belongs to the TNF superfamily involved in the regulation of a wide spectrum of biological processes including cell survival, proliferation, differentiation, and cell death
Tuberous sclerosis 1—*TSC1*	Rare sequence variants (https://gene.sfari.org/database/human-gene/, accessed August 5th 2022)	Tumor suppressor. Interacts with TSC2 generating a protein complex which negatively regulates mTORC1 signaling, the main regulator of anabolic cell growth. Co-chaperone function inhibiting the ATPase activity of Hsp90
Tuberous sclerosis 2—*TSC2*	Rare sequence variants (https://gene.sfari.org/database/human-gene/, accessed August 5th 2022)	Tumor suppressor. Interacts with TSC1 and form the TSC protein complex which controls cellular growth
Uncoupling protein 2—*UCP2*	Increased gene expression (201)	Member of mitochondrial anion carrier protein family with important role in prevention of oxidative stress

## Immune-gut axis in ASD

Microbiome composition differs in ASD individuals compared with typically developing ones (203), and numerous reports describe gut dysbiosis associated with metabolic imbalance and immune dysregulation in ASD. The question to be answered is if the gut dysbiosis is a consequence or a cause of ASD? Recent studies tried to answer this question and to decipher the genetic factors that intermingle in ASD with the triad microbiota-metabolism-immune system (122, 204, 205). The gastrointestinal system (GI) is intricately connected with the immune system, and chronic inflammatory processes are suggested to contribute to the GI symptoms which frequently occur in individuals with ASD (206–208). Intestinal barrier dysregulations trigger pro-inflammatory processes and release pro-inflammatory cytokines and activated monocytes. These can reach the blood-brain barrier through the bloodstream, thus contributing to neuroinflammation (94, 209).

A whole-exome sequencing study in a group of ASD children with GI symptoms performed by Liu et al. revealed significantly increased single nucleotide variants (SNVs) distribution in genes involved in innate immune response, glycosylation and retrograde axonal transport. The identified SNVs correlated with the microbiome composition and the obtained data emphasized that the interaction of host genetics and gut microbiome may induce immune deregulation and metabolism inference (204). In another recent study, Wang et al. showed that VFGM gene imbalance may reflect dysregulation of the gut's immune function in ASD, and suggests common mechanisms such as gut inflammation and gut microbiota influencing neuroinflammation in ASD (118).

RNA sequencing (RNAseq) performed on GI tissue from ASD children detected differentially expressed transcripts (DETs) regulating immune and inflammatory response (210). T cell receptor (TCR) activation was mainly depicted in Th1 and Th2 arms including multiple signaling pathways (e.g., iCOS-iCOSL TREM1, NF-kappaB, Toll-like receptor signaling). Most of the identified DETs include metabolic pathways (tryptophan, serotonin and melatonin degradation, ketogenesis, ketolysis, oxidative phosphorylation), endocrine pathways (activation of pregnane X receptor, retinoid X receptor, and farnesoid X receptor) and mitochondrial dysregulation (210). Tryptophan degradation was signaled as deficient in ASD, being pointed out as a possible biomarker (211). A substantial number of transcripts in the mitochondrial pathways were found upregulated in GI-ASD, namely nicotinamide adenine dinucleotide (NADH) dehydrogenase, NADH:ubiquinone oxidoreductase core subunit 4L *(ND4L)*, cytochrome B (*CYB)*, ubiquinol-cytochrome c reductase core protein 1 (*UQCRC1)*, and cyclooxygenases (*COX*)—*COX1, COX2, COX5B, COX6A1, COX6B2* (210). TNF-α transcript and other additional TNF-related transcripts were found up-regulated in both GI and peripheral lymphocytes in ASD children (210, 212) contributing to the mitochondrial dysfunction in the context of gut inflammation. Therefore, with regard to the immune-gut axis in ASD, some important points should be underlined. Taking into account that immune dysregulation, immune and autoimmune diseases were described in ASD individuals or families (213), and that gut microbiota imbalance has an important impact on both immune and nervous systems, the interactions of brain, gut and immune system may contribute to ASD pathophysiology (118, 122, 205).

Gut microbiota, has an intense communication with many other systems so that the “Immune-Gut-Brain axis” seems to have an important impact in neurodevelopmental disorders.

**Figure 3:**
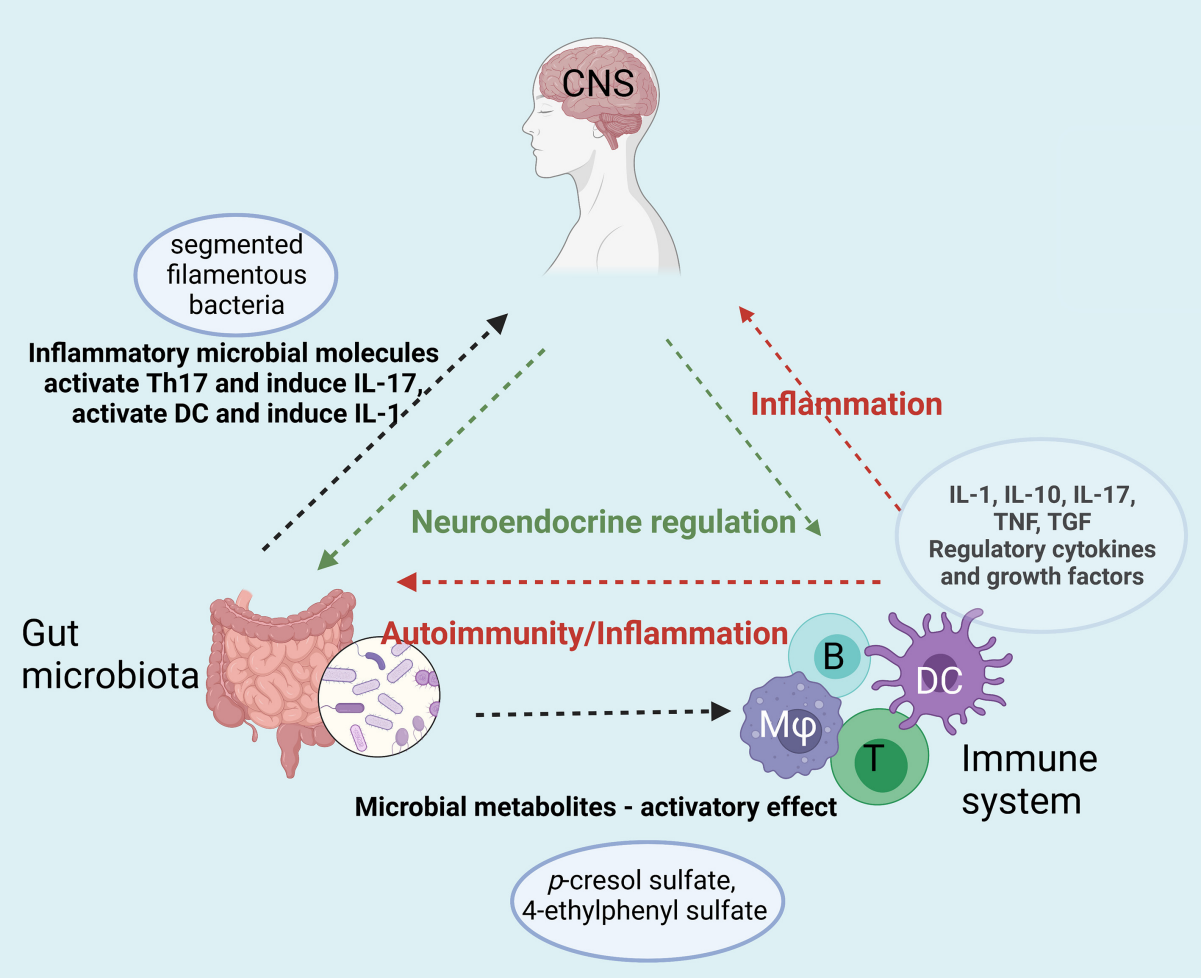
Immune-Gut-Brain network that converge toward sustaining neuroinflammation in ASD. Gut microbiota enhances the inflammatory status by specific microbial metabolites (e.g., p-cresol sulfate, 4-ethyphenyl sulfate) that activate both innate (dendritic cells, macrophages) and adaptive immune cells (T and B lymphocytes). The enhanced pro-inflammatory cytokine secretion and the auto-antibodies generated upon this hyperactivation act on both digestive system and on the central nervous system sustaining the neuroinflammation. Moreover, gut microbiota enhances the inflammatory status by specific microbial molecules that append mainly to the segmented filamentous bacteria that activate Th17 and induce IL-17 secretion [B-B, lymphocyte; T-T, lymphocyte; DC, dendritic cell; (Mφ), monocyte/macrophage]. Created with BioRender.com.

A diagram of the putative interplay of the immune system-gut microbiota and CNS dysregulation is presented in Figure 3.

## Immune-related epigenetics of ASD

Epigenetics has followed the research foot-steps of genomics and proteomics and, in recent years, it expanded in various fields, from cancer (214) to autoimmune diseases (215), aiming to explain complex biological processes. The epigenetic machinery consists of a multitude of molecules and processes that are interlinked. DNA methylation/demethylation, histone modification, non-coding RNAs [e.g., long non-coding RNAs (lncRNA), circular RNA, miRNA] are just a few molecular players that can induce heritable phenotypic changes not affecting *per se* the DNA sequence. If epigenetics disrupts gene expression it can lead to various major human pathologies and epigenetics molecules can be markers for patient's diagnostic, stratification, and/or therapy (216).

Epigenetic changes are proposed ASD contributors, as mediators at the crossroads between environmental factors and genome during development (217, 218). DNA methylation is essential for brain development, being one of the most frequently studied epigenetic regulation mechanism (219, 220). Studies of genome-wide DNA methylation patterns were performed on various tissues from ASD individuals, such as blood (221), buccal epithelium (220, 222, 223), brain (218, 224–226), cord blood and placenta (227–229). Each of the above-mentioned tissues has advantages, such as accessibility for peripheral tissues or a more homogeneous cellular composition for buccal epithelium, as well as limitations, such as extremely reduced sample sizes for post-mortem brain tissue or cell heterogeneity for peripheral blood (220, 222). Jangjoo et al. compared the genome-wide DNA methylation (DNAm) profiles in blood samples from children with ASD and typically controls and found no significant differences between the two groups (221). However, a subset of ASD children had a DNAm pattern distinct from the rest of ASD children and from controls. These methylation differences were mainly associated with deregulations of immune cell type circulating proportions, although ASD risk genes were also differentially methylated (221). Several studies reported brain DNA methylation alterations, in areas such as prefrontal cortex, temporal cortex, cingulate gyrus, subventricular zone, and cerebellum, in ASD individuals compared to controls (218, 224–226). These data indicate the existence of common differentially methylated regions in ASD thus bringing new evidence that support the role of epigenetic changes in ASD pathophysiology and contribute to the discovery of new candidate genes (218, 224–226). The perinatal tissues, such as placenta and cord blood revealed interesting data regarding epigenetic changes in early developmental. Due to its distinct pattern of DNA methylation, similar to oocytes and preimplantation embryos (230), placenta proved to be a promising tissue for studying DNA methylation changes in ASD (217, 228, 231). The detection of differentially methylated regions in ASD, as epigenetic markers, highlight the value of DNA methylation investigation prior to symptom onset and bring novel insights for early recognition and therapeutic approaches.

Non-coding RNAs are other epigenetic players recently identified in ASD. Changes in the expression level of lncRNAs were reported in ASD as summarized in Table 3. Among epigenetic regulators altered in ASD, several miRNAs involved in the development of immune system and immune responses were reported, along with those regulating major pathways like PI3K/Akt/mTOR and epidermal growth factor receptor (EGFR) intracellular signaling (232).

**Table 3:** Non-coding RNAs found altered in ASD.

**Symbol (Name)**	**Expression sites (https://gtexportal.org/home/)**	**Expression level**	**References**
RP11-466P24.2 (ENST00000502589)	Various tissues, including white blood cells and brain	Decreased	(233)
SYP Antisense RNA 1–SYP-AS1 (ENST00000527880)	Brain and adrenal tissue	Decreased	(233)
Syntaxin Binding Protein 5 Antisense RNA 1–STXBP5-AS1 (ENST00000433499)	Various tissues, including immune cells and nervous system; found in breast cancer	Decreased	(233)
Interferon Gamma Antisense RNA 1–IFNG-AS1 (ENSG00000255733)	Various tissues, including immune cells and nervous system; found in autoimmune-diseases	Decreased	(234)
AK128569 (uc001mff.1)	Various tissues, including immune cells, EBV transformed lymphocytes	Increased	(233)
Synaptotagmin 9 Antisense RNA 1–SYT9AS, CTD-2516F10.2 (ENST00000504206)	Various tissues, including basal ganglia	Increased	(233)
Moesin Pseudogene 1 Antisense RNA 1–MSNP1AS (ENSG00000251593)	Various tissues, including immune cells, nervous system, endocrine system, and various internal organs	Increased	(235, 236)
Ribosomal Protein S10 Pseudogene 2 Antisense RNA 1–RPS10P2-AS1	Fetal temporal cortex and adult peripheral blood	Increased	(237)
Long Intergenic Non-Protein Coding RNA 693–LINC00693	Various tissues, including immune cells and nervous system; found in autoimmune-diseases	Increased	(238)
Long Intergenic Non-Protein Coding RNA 689–LINC00689	Various tissues, including lymph nodes, placenta, testis	Increased	(238)
Maternally Expressed 3–MEG3 (ENSG00000258663)	Various tissues, including endocrine system, nervous system	Increased	(239)
Nuclear Paraspeckle Assembly Transcript 1–NEAT1 (ENSG00000245532)	Various tissues, including immune cells, nervous system, endocrine system, and various internal organs	Increased	(240)
Taurine Up-Regulated 1–TUG1 (ENSG00000253352)	Various tissues, including immune cells, lymph nodes and nervous system	Increased	(240)
SH3 And Multiple Ankyrin Repeat Domains 2 Antisense RNA–SHANK2 AS (ENSG00000226627)	Low expression in various tissues	Increased	(241)

Several studies reported the involvement of small RNA both in central nervous and the immune system regulation. Down-regulation of hsa_can_1002-m was observed in the cerebral cortex of ASD individuals. This miRNA is predicted to modulate activity of EGFR and FGF receptor (FGFR) signaling pathways which are involved in brain development and inflammatory/immune processes (36).

A recent study using small RNAseq analysis on lymphoblastoid cell lines derived from ASD children identified a series of miRNAs with dysregulated expression. The predicted targeted genes of these miRNAs are involved in important pathways, such as MAPK signaling, cytokine–cytokine receptor interaction, spliceosome, calcium signaling, and WNT signaling. Further expression analysis of genes targeted by two selected miRNAs, miR-181a-5p and miR-320a, which were down-regulated in ASD individuals compared to controls, showed dysregulation of genes involved both in the central nervous and immune system, namely AKT serine/threonine kinase 2 (*AKT2*), AKT serine/threonine kinase 3 (*AKT3*), *TNF*-α, calcium/calmodulin dependent protein kinase II alpha *(CAMK2A)*, and beta *(CAMK2B)* (242).

Overall, the epigenetic changes in ASD can contribute to altered gene expression of targeted genes. The epigenetic alterations and the subsequent transcriptional changes converge functionally to known ASD pathways (243). Moreover, epigenetic modification in response to environmental factors open new avenues toward understanding this complex entity and possibly to therapeutic interventions (244).

## Discussion

ASD is a neurodevelopmental disorder characterized by impairment of social interaction and communication, as well as restricted interests and stereotyped and repetitive behavior patterns (1).

ASD has a multifactorial etiology. The genetic and environmental risk factors interplay has detrimental consequences primarily on the central nervous systems; other systems such as the immune and digestive system are also dysregulated. Sequence variants have been reported in numerous genes, however there are still no “autism genes” but “brain-genes” (https://staging.spectrumnews.org, Accessed September 14th 2022). The epigenetic changes observed in ASD can contribute to the altered gene expression of many targeted genes. Moreover, the epigenetic changes in response to environmental factors open new avenues toward understanding this complex entity and possibly to therapeutic interventions (243, 244).

Among the environmental factors that increase ASD risk, those acting in the prenatal period and in the maternal environment in which the fetus is developing, are regarded as the most harmful. Maternal immune activation, brain-reactive antibodies and autoimmunity are proposed contributors to ASD pathophysiology (32, 35, 111–113).

Both genetic and epigenetic factors can contribute to the immune dysregulations in ASD. Early studies have shown that PBMCs from ASD children are characterized by an excess of pro-inflammatory cytokines, their function being dysregulated by the lack of anti-inflammatory mechanisms (245). Other studies have also provided evidence supporting the role of IL-17A in neuro-inflammatory processes; in addition, monocytes, B lymphocytes, and neutrophils produce oxidative and inflammatory mediators (43, 44, 52). Recently NK cells joined the immune arsenal proposed to contribute to neuro-inflammation in ASD (246). Among the mediators of immune dysregulation, cytokines are considered to have a major role. IL-1 is involved in various neuronal physiological pathways, e.g., modulation of neural plasticity, synaptic plasticity, neuronal calcium signaling, thus has been in the spot light of ASD research (66–68, 247). Other immune molecules, such as IL-6, TNF-α, IL-8, IL-31, IL-16, and IL-12p40, also contribute to the inflammatory milieu in ASD (45, 78, 80). Therefore, the entire immune system, with its complex network of molecules and immune cells, is involved in the dysregulation of innate and adaptive immune responses in ASD. Oxidative stress is a direct consequence of immune dysregulation; this phenomenon has been reported in many acute and chronic brain disorders (176, 186). ROS are involved in neuroinflammation, and although the innate immune cells are the main source of ROS, adaptive immune cells T and B cells also contribute to the oxidative stress observed in ASD (178–180). The imbalance generated by the increased ROS production and decrease of anti-oxidant regulatory mechanisms leads to oxidative stress. Oxidative stress, further dysregulates mitochondrial physiology, metabolic pathways, gut homeostasis, and other immune responses, contributing to neuroinflammation (177).

Further research on the proinflammatory molecular arsenal, with its main players, the cytokines, may contribute to establishing ASD immune endophenotypes, and consequently driving current and future ASD therapeutic interventions to more personalized approaches (248, 249). Moreover, neurobehavioral symptoms severity appears to correlate with the extent of systemic immune alteration (41). This opens new perspectives for prediction of clinical evolution and therapeutic guidance. The field of biological biomarkers in ASD is promising, with current markers awaiting further validation and new markers being continuously discovered. Panels of immune molecules have the potential to become robust ASD-related biomarkers that may aid the diagnosis, patient stratification, and monitoring (50).

## Conclusions

Significant discoveries regarding the molecular and immunological features of individuals with ASD have been reported during the past years. Multiple studies have been focused on the genetic architecture of ASD, known to be complex and highly heterogeneous. Gathering prenatal and early post-natal comprehensive information, individualized profiling can be done in order to advance the clinical care toward precision medicine approaches in ASD. Identification of immune-related genes and their interactions on several levels has shown that the main immune dysregulation resides in the inflammatory area impacting early neuronal development and function. Gene encoding immune molecules, such as cytokines and several other immune-related elements involved in antigen processing, oxidative stress and mitochondrion function converge toward the overall pro-inflammatory status of ASD.

A better understanding of the immune-mediated pathways and their impact on many other biological processes and systems, such as metabolism, the endocrine and gastrointestinal systems, may contribute to the discovery of new therapeutic targets in ASD, ultimately aiming to improve the quality of life of ASD individuals.

## Author contributions

MN and AE conceived, designed the structure, and contributed to the writing of the paper. AA, SP, and MB contributed to the writing of the paper and the preparation of figures. MN, AE, and AA edited the paper. All authors approved the manuscript.

## Funding

The research leading to these results has received funding from the EEA Grant 2014-2021, under the project contract no 6/2019. Article processing charge (APC) was funded from the same grant.

## Conflict of interest

The authors declare that the research was conducted in the absence of any commercial or financial relationships that could be construed as a potential conflict of interest.

The reviewer MV declared a past co-authorship with one of the authors MN to the handling editor.

## Publisher's note

All claims expressed in this article are solely those of the authors and do not necessarily represent those of their affiliated organizations, or those of the publisher, the editors and the reviewers. Any product that may be evaluated in this article, or claim that may be made by its manufacturer, is not guaranteed or endorsed by the publisher.

## Glossary

ADA, adenosine deaminase; AKT2, AKT serine/threonine kinase 2; AKT3, AKT serine/threonine kinase 3; ApaI, abbreviation of a *VDR* gene polymorphism; ASD, autism spectrum disorder; BAFF, B-cell activating factor; BsmI, abbreviation of a *VDR* gene polymorphism; CAMK2A, calcium/calmodulin dependent protein kinase II alpha; CAMK2B, calcium/calmodulin dependent protein kinase II beta; CCR3, C-C motif chemokine receptor 3; CD, cluster of differentiation; Cj1137c, glycosyltransferase; COX, cyclooxygenases; CSF, cerebrospinal fluid; CSTB, Cystatin B; CXCR, C-X-C motif chemokine receptor; CYB, cytochrome B; DETs, differentially expressed transcripts; DNA, deoxyribonucleic acid; DNAm, DNA methylation; DRP1, dynamin 1 like; EGFR, epidermal growth factor receptor; FGF, fibroblast growth factor; FGFR, fibroblast growth factor receptor; FIS1, fission, mitochondrial 1; FokI, abbreviation of a *VDR* gene polymorphism; FRα, folate receptor alpha; GBS, group B streptococcus; GI, gastrointestinal system; GM-CSF, granulocyte-macrophage colony-stimulating factor; GSTA1, glutathione S-transferase alpha 1; GSTM1, glutathione S-transferase mu 1; GSTP1, glutathione S-transferase pi 1; GSTs, glutathione transferases; GSTT1, glutathione S-transferase theta 1; H2O2, hydrogen peroxide; HIGD2A, HIG1 hypoxia inducible domain family member 2A; HLA, human leukocyte antigen; HLA-A, major histocompatibility complex, class I, A; HLA-DRB1, HLA class II histocompatibility antigen, DRB1 beta chain; HOCl, hypochlorous acid; HSP70i, inducible heat shock protein 70; IFN, interferon; Ig, immunoglobulins; IL, interleukin; iNOS, inducible nitric oxide synthase; JAK1, Janus kinase 1; kfiC, glycosyltransferase; LCL, lymphoblastoid cell line; lncRNA, long non-coding ribonucleic acid; LPS, lipopolysaccharide; MAPK, mitogen-activated protein kinase; MCP, macrophage chemoattractant protein; MFN1, mitofusin 1; MFN2, mitofusin 2; MHC, major histocompatibility complex; miRNA, micro ribonucleic acid; MPO, myeloperoxidase; MRC1, mannose receptor C-type 1; mRNA, messenger ribonucleic acid; mTOR, mammalian target of rapamycin kinase; NADH, the reduced form of nicotinamide adenine dinucleotide; NADPH, nicotinamide adenine dinucleotide phosphate; ND4L, NADH:ubiquinone oxidoreductase core subunit 4L; NDDs, neurodevelopmental disorders; NF1, neurofibromin 1; NK, natural killer; NO, nitric oxide; NOSs, NO synthases; NOX, nicotinamide adenine dinucleotide phosphate oxidase; NR4A1, nuclear receptor subfamily 4 group A member 1; OPA1, OPA1 mitochondrial dynamin like GTPase; PBMCs, peripheral blood mononuclear cells; PRKAA2, protein kinase AMP-activated catalytic subunit alpha 2; PTEN, phosphatase and tensin homolog; RNA, ribonucleic acid; RNAseq, RNA sequencing; RNS, reactive nitrogen species; ROS, reactive oxygen species; S6K1, ribosomal protein S6 kinase beta-1; SCID, severe combined human immune deficiency; SNVs, single nucleotide variants; SOD, superoxide dismutase; STAT, signal transducer and activator of transcription; TaqI, abbreviation of an *VDR* gene polymorphism; TCR, T cell receptor; TGF, transforming growth factor; Th, helper cells; THRIL, TNF and HNRNPL related immunoregulatory long non-coding RNA; TLR4, toll like receptor 4; TNF, tumor necrosis factor; Tnfsf13b, TNF superfamily member 13b gene; mTORC1, mechanistic target of rapamycin complex 1; TSC1, TSC complex subunit 1; TSC2, TSC complex subunit 2; UCP2, uncoupling protein 2; UQCRC1, ubiquinol-cytochrome c reductase core protein 1; VDR, vitamin D receptor; VFGM, virulence factor-related gut microbiota; wlaN, beta-1,3 galactosyltransferase; ZNF322, zinc finger protein 322.
